# tRNA Derived smallRNAs: smallRNAs Repertoire Has Yet to Be Decoded in Plants

**DOI:** 10.3389/fpls.2017.01167

**Published:** 2017-07-25

**Authors:** Gaurav Sablok, Kun Yang, Rui Chen, Xiaopeng Wen

**Affiliations:** ^1^Finnish Museum of Natural History Helsinki, Finland; ^2^Department of Biosciences, Viikki Plant Science Center, University of Helsinki Helsinki, Finland; ^3^Key Laboratory of Plant Resources Conservation and Germplasm Innovation in Mountainous Region, Ministry of Education, Institute of Agro-Bioengineering and College of Life Sciences, Guizhou University Guiyang, China; ^4^Tianjin Institute of Agricultural Quality Standard and Testing Technology, Tianjin Academy of Agricultural Sciences Tianjin, China

**Keywords:** smallRNAs, tRNAs, microRNAs, functional genomics, stress

## Abstract

Among several smallRNAs classes, microRNAs play an important role in controlling the post-transcriptional events. Next generation sequencing has played a major role in extending the landscape of miRNAs and revealing their spatio-temporal roles in development and abiotic stress. Lateral evolution of these smallRNAs classes have widely been seen with the recently emerging knowledge on tRNA derived smallRNAs. In the present perspective, we discussed classification, identification and roles of tRNA derived smallRNAs across plants and their potential involvement in abiotic and biotic stresses.

## smallRNAs: Post-Transcriptional Check Points

Post-transcriptional regulation represents an integrated network of array of RNAs, among which regulatory RNAs play a major role in deciphering and regulating the functional omics. Rise of next generation sequencing approaches have widely elucidated several class of regulatory RNAs, often categorized into small non-coding RNAs and long non-coding RNAs (Heo et al., 2013). The functional role of these regulatory RNAs has been well described in plant genomics and has conserved or distinct functional roles in plants either through the RNA directed DNA methylation (RdDM pathway) or by epigenetically silencing the transposable elements (Chan et al., 2004). Swathing information on small non-coding RNAs have been revealed across a wide range of plant species focussing on most abundant class of smallRNAs – microRNAs (Zhang et al., 2006). Origin of microRNAs has been attributed to several key events such as the coordination of the DICER-like proteins (DCL-1) and methylated *HEN*1 for microRNA biogenesis and targeted transcriptional and translational repression through cleavage site interactions. Coordinated regulatory activities and interaction networks of these smallRNAs classes have been previously shown to play a key role in understanding the plant functional and developmental genomics (Rubio-Somoza and Weigel, 2011; Meng et al., 2011; Li and Zhang, 2016). With the rapid development in the next generation sequencing technologies and miRNAs moving to the single cell miRNAs transcriptome (Faridani et al., 2016), a new class of smallRNAs, tRNA derived smallRNAs (Hsieh et al., 2009), which have been explored widely in animals have now recently started gaining substantial importance in plant genomics with recent evidences showing their canonical interactions with AGO1 and TE like microRNAs (Alves et al., 2017; Martinez et al., 2017).

Although the biogenesis of these tRNA derived smallRNAs, cleavage efficiency and target accessibility has been addressed with limited reports in plants, signatures of association of tRNA derived smallRNAs with AGO proteins in particular AGO1, which also loads miRNAs revealed their role in the transcript repression, were also observed for tRNA derived smallRNAs (Loss-Morais et al., 2013; Martinez et al., 2017). Alongside, this class of smallRNAs has been shown to be involved in translation repression in *Cucurbita maxima* where phloem specific tRNAs fragments have been shown to interfere with ribosomal activity and represses translation (Zhang et al., 2009). Interestingly, recent comparative analysis revealed hints toward the biogenesis of these tRNA derived smallRNAs independent of DICER-like proteins (Alves et al., 2017), however, reduced abundance of tRNA derived smallRNAs has been observed in *dcl1* double mutants (Martinez et al., 2017). Using both *dcl1* and *ago1* single mutants, specific reduction of the tRNAs derived smallRNAs was observed as compared to miRNAs, which concludes the involvement of AGO1 in tRNA derived smallRNAs biogenesis pathway (Martinez et al., 2017). Taking into account this new emerging class of smallRNAs with relatively less explored associations with AGO proteins and potential roles in transcriptional and translational repression, it is an intriguing question to address in plant genomics the role and functional diversity of tRNAs derived smallRNAs and their roles in context to most widely profiled class of miRNAs.

## tRNA Derived smallRnas: Small Non-Coding Functional Beast

tRNA derived smallRNAs represent a class of 19-mer smallRNAs, which have been previously widely demonstrated in humans and play an intriguing role in regulating the gene expression post-transcriptionally (Sobala and Hutvagner, 2011). In humans, their interactions with the other established class of small non-coding RNAs such miRNAs and siRNAs have been widely demonstrated (Garcia-Silva et al., 2012). In plants, however, the detection and possible association of tRNA derived smallRNAs (Nowacka et al., 2013) is still lacking with only few reports indicating the roles of tRNAs derived smallRNAs in tissues, embryogenic callus (Chen et al., 2011), phloem (Zhang et al., 2009) and recently in pollens (Martinez et al., 2017). tRNA derived smallRNAs classification and nomenclature in plants have been correlated with 5′, 3′ and CCA proximities and have been length classified as 19–25 nt (Loss-Morais et al., 2013; Alves et al., 2017). Taking into account the length variations in correlation with abundance, so far observed length variations were found to be between 19 and 25 nt, while majority of them belonging to 5′ 19-nt and has been seen conserved across dicot (*Arabidopsis thaliana*-Gly^UCC^), *Zea mays* (C4 species) and evolutionary conserved moss (*Physcomitrella patens*) (Alves et al., 2017; Martinez et al., 2017) except monocot (*Oryza sativa*- Ala^AGC^), where 25-nt abundance was seen as the most dominant length of these smallRNAs (Chen et al., 2011). Previously observed correlation of length and corresponding abundances of tRNA derived smallRNAs are also supported by recent observations of Martinez et al. (2017), which highlighted the abundance of the 19-nt tRNAs derived smallRNAs as a part of the 5′ tRNA processing in pollens of *Arabidopsis thaliana*. However, across polyploids such as *Triticum aestivum*, abundance of 21-nt with most of them originating from Val^CAC^ has been observed (Wang Y. et al., 2016). Despite being conserved in patterns of distribution across monocots and dicots, *Oryza sativa* AGO reveals high abundance of specific Arg^CCT^ and Arg^TCG^ tRNA derived 19-nt smallRNAs as compared to the Ala^AGC^ (Alves et al., 2017). Difference in these patterns of length abundances might be due to species ploidy or due to the cleavage asymmetry and role of anticodon loop required for tRNA processing (Wang Y. et al., 2016). Chen et al. (2011) established the first report on the identification of tRNAs derived smallRNAs from meristem associated smallRNAs sequencing and differential regulation of 5′Ala^AGC^ and Pro^CGG^ in callus and leaves, which is also supported by the recent reports in *Arabidopsis thaliana* addressing the tissue specificity of tRNAs derived smallRNAs (Alves et al., 2017). Recently pollen specific accumulation of tRNAs derived smallRNAs (Ala^AGC^) has been found and interestingly using the *dcl1* mutants they showed the interactions of these smallRNAs with transposable elements (TE) in *Arabidopsis thaliana* (Martinez et al., 2017).

Although the biogenesis of tRNA-derived smallRNAs has not been functionally elucidated, recent reports indicate toward an independent processing machinery, which doesn’t have functional interplay of DICER-like proteins (Alves et al., 2017). This is in contrast to the recent report of Martinez et al. (2017) using the *dcl1* double mutants revealing the association of DCL-1 proteins and tRNAs derived smallRNAs. Although the functional processing machinery might be different but their association with the AGO1, 2, 4 and 7 (*ARGONAUTE*) proteins have been experimentally verified using the immunoprecipitated AGO proteins (AGO-IP) (Loss-Morais et al., 2013; Alves et al., 2017; Martinez et al., 2017). Moreover, length variations also affects the association of tRNA derived smallRNAs to AGO proteins. Alves et al. (2017) demonstrated the association of the 19- and 20-nt tRNA derived smallRNAs with AGO2 and AGO5 whereas AGO4 was found to be abundant with 19-, 24-, and 25-nt respectively.

It is worth to mention that not only tRNA derived smallRNAs biogenesis pathways showed independency with respect to microRNAs, the presence of terminal 5′ nucleotide is also interestingly different, whilst tRNA derived smallRNAs preferred to have a G as 5′ terminal as compared to microRNAs, which prefer to have either a U or A as the 5′ terminal nucleotide (Loss-Morais et al., 2013). These observations are similar to the recent observation in fungal pathogen *Phytophthora sojae* (Wang Q. et al., 2016) suggesting the conservation pattern of these preference for 5′ terminal nucleotides. Conservation of cleavage site analysis provides support that the origin of these tRNA derived smallRNAs is not a random exonucleolytic digestion of the tRNAs precursors (Alves et al., 2017). Interestingly, target site analysis revealed the unusual target cleavage at multiple sites in tRNA-derived smallRNAs as compared to the single center binding sites in the miRNAs and siRNAs (Wang Y. et al., 2016). However, using the PARE-seq data, pollen specific tRNAs derived smallRNAs describe the mode of cleavage site action canonical to miRNAs (Martinez et al., 2017). Recent investigations using the nucleotide composition analysis across the cleavage sites revealed preferable enrichment of U across the cleavage site for both 5′ and 3′ tRNAs derived smallRNAs (Wang Y. et al., 2016).

Organelle genomes play an important role in response to abiotic stress and control the photosynthetic regulatory activities under abiotic stress. Although being small in size as compared to the nuclear genome, recent reports reveal a large percentage (25%) of the tRNAs derived smallRNAs derived from organelle genomes in particular chloroplast (Cognat et al., 2017). The observed abundance of the plastid encoded tRNAs supports the previous dynamic regulation of chloroplast encoded Tyr^GTA^ during the ASGV infection in *Malus* x *domestica* (Visser et al., 2014). Interestingly, the profiled tRNAs derived smallRNAs population represented both forms tRF-5D (due to a cleavage in the D region) and tRF-3T (via a cleavage in the T region) (Cognat et al., 2017). However, specific enrichment of the tRF-5D was seen with AGO1 immunoprecipitation libraries (Cognat et al., 2017). Although high abundance of the plastid encoded tRNAs smallRNAs was observed, however, they were found to be localized outside organelle. Taking into account these reports, it is yet to be addressed the transport mechanism and the functional role of the plastid encoded tRNAs derived smallRNAs.

Although been recently discovered, genome wide implications on the role of these tRNA derived smallRNAs has been shown in abiotic and biotic stress (Thompson et al., 2008; Asha and Soniya, 2016; Wang Q. et al., 2016). Initial reports indicating the possible involvement of these tRNA derived smallRNAs such as 5′ tRF of Asp^GTC^, and 3′ CCA tRFs of Gly^TCC^, which were found to be over-expressing in phosphate starvation and drought (Hsieh et al., 2009; Loss-Morais et al., 2013). A compiled list of the tRNA derived smallRNAs and their potential involvement in stress has been presented as **Table 1**. Interestingly, Alves et al. (2017) highlighted the role of RNS1 (*RIBONUCLEASE 1*) using a T-DNA insertion line (*rsn1-*1, D2lk1087165C) with possible involvements in the tRNA derived smallRNAs biogenesis. RNS1 belongs to the ribonuclease T_2_ family, whose another member s-RNASE has been shown to be the key member involved in self-incompatibility (Bariola et al., 1999) and has been shown to be mainly regulated under phosphate (Pi) starvation and anthocyanin regulation (Bariola et al., 1999). Potential role of tRNA derived smallRNAs has also been found ruing the heat stress in polyploids such as *Triticum aestivum*, where the observed tRNAs derived smallRNAs population was found to be predominantly coming from the mature arms of tRNAs as compared to the nascent transcripts in polyploids (Wang Y. et al., 2016). Interestingly, diversification of the functional tRNA derived from the same amino acid was seen in *Triticum aestivum*, with Met^CAU^ displayed lower expression as compared to the other processed isotype, from the same isoacceptor, which showed up-regulation during the heat stress (Wang Y. et al., 2016). This along with the increased cleavage of 3′ ends during the heat stress confers the dynamic changes in the tRNA derived smallRNAs pool during the abiotic stress.

**Table 1:** tRNA derived sequences and their revealed roles in abiotic and biotic stress.

tRNA derived sequence	Stress/Development	Reference
Ala^AGC^	Drought/Salt	Loss-Morais et al., 2013
Arg^CCT^	Drought	Loss-Morais et al., 2013; Alves et al., 2017
Arg^TCG^	Drought	Loss-Morais et al., 2013
Gly^TCC^	Drought	Loss-Morais et al., 2013
Asp^GTC^	Phosphate starvation	Hsieh et al., 2009
Gly^TCC^	Phosphate starvation	Hsieh et al., 2009
Arg^CCT^	Cold	Alves et al., 2017
Arg^TCG^	Oxidative stress	Alves et al., 2017
Tyr^GTA^	Oxidative stress	Alves et al., 2017
IIe^AAT^	Pathogen	Wang Q. et al., 2016
Arg^ACG^	Pathogen	Wang Q. et al., 2016
Ala^CGC^	Pathogen	Asha and Soniya, 2016
Val^CAC^, Thr^UGU^, Tyr^GUA^, Ser^UGA^	Heat stress	Wang Y. et al., 2016

Systematic profiling of smallRNAs during Apple stem grooving virus (ASGV) infection in *Malus* x *domestica* revealed a large proportion of tRNA derived smallRNAs. Interestingly, their association with miRNAs, tasiRNAs, phasiRNAs were not observed (Visser et al., 2014). Visser et al. (2014) reported 33-nt tRNA derived smallRNAs as the most abundant ones with the most abundant being 5′ tRNA-half originating from Asp^GTC^. As previously revealed during the abiotic stress, differential regulation of tRNAs derived smallRNAs and also smallRNAs overlapped by tRNAs were found to be differentially regulated in ASGV infected samples, one of the abundant tRNAs (Tyr^GTA^) was found altering the sRNAs arrangement in ASGV-infected samples. Strikingly, the infections states showed inverse correlations of fragment types (those originating from the 3′ and extending into the variable regions and those originating from the central stem region of the tRNAs). This arguable contrasting pattern might hint toward the co-existence of the separate biogenesis pathways.

In plants, pathogen associated immunity is controlled through the microbial or pathogen associated molecular patterns (MAPS or PAMPs), which upon the pathogen or the microbial infection trigger the defense response through up-regulation of defense related genes as a part of plant immunity. Asha and Soniya (2016) demonstrated the involvement of the tRNAs derived smallRNAs in regulating the expression patterns of defense related genes during *Phytophthora capsici* infection in Black Pepper (*Piper nigrum* L.). Interestingly the dominance of 5′ tRNA derived smallRNAs was found among the observed tRNAs population, which supports that in plants the major class of these smallRNAs is represented by 5′ tRNAs (Alves et al., 2017; Martinez et al., 2017). Experimental confirmation of 5′ Ala^CGC^ target sites on Non-expresser of pathogenesis related protein (NPR1) confirmed the repression of the NPR1 during the pathogen infection (Asha and Soniya, 2016). Above confirmatory results establish that tRNA derived smallRNAs indeed supresses the expression pattern of target genes as previously observed in Zhang et al. (2009), where the phloem specific tRNAs were found to interact with the ribosomal activity thus leading to translational repression.

A key message from Loss-Morais et al. (2013) indicated the association of these tRNAs derived smallRNAs to AGO2, which is an AGO of unknown function and has been shown to have relatively higher levels during biotic infections (Zhang et al., 2011). However, recent reports of Wang Q. et al. (2016), showed contrasting association of the tRNA derived smallRNAs to AGO1 in pathogenic infection. Taking into account these observations, it can be presumed that association of the tRNA-derived smallRNAs might be species or organism specific in response to abiotic or biotic stress. However, taking together the AGO1 associations and recent reports of AGO1 and DCL1 association using the *dcl1* double mutants, it is noteworthy to highlight that these classes of smallRNAs has regulatory roles, which are yet to be discovered in plants, which can add to the understanding of the plant functional genomics. It is worthwhile to conclude that plants harbor the most abundant pool of tRNAs with clearly distinct nuclear and organelle tRNAs and therefore these classes of tRNAs, their abundance, profiling and genetic interactions with the cognate targets would expand understanding of the complex RNA’ome in plants.

## Author Contributions

GS conceived and drafted the manuscript, KY, RC, and XW provided revisions to the MS.

## Conflict of Interest Statement

The authors declare that the research was conducted in the absence of any commercial or financial relationships that could be construed as a potential conflict of interest.
